# Humor as a Multifaceted Resource in Healthcare: An Initial Qualitative Analysis of Perceived Functions and Conditions of Medical Assistants’ Use of Humor in their Everyday Work and Education

**DOI:** 10.1007/s41042-022-00074-2

**Published:** 2022-10-13

**Authors:** Julia Raecke, René T. Proyer

**Affiliations:** 1 Federal Institute for Vocational Education and Training, Division 1.4 - Competence Development, Bonn, Germany; 2 Department of Psychology, Martin-Luther University Halle-Wittenberg, Halle, Germany

**Keywords:** Humor, Healthcare, Medical Assistants, Resources, Comic Style Markers, Positive Education

## Abstract

It has been argued that humor serves as a crucial resource for healthcare professionals (HCPs). For example, they can use it to cope with work stress, to build relationships with patients, and/or to educate medical students and residents—to name but a few functions. However, empirical studies on the importance of humor among HCPs are still scarce. Existing studies primarily focus on nurses and physicians and rarely distinguish between different humor styles (e.g., light and dark). Based on qualitative interviews with 14 German Medical Assistants (MAs), we investigate the potential of humor as a resource for MAs’ work and education. We focus on the perceived functions and conditions of MAs’ successful use of humor as well as the role of humor styles (i.e., comic styles). Results indicate various functions of humor in MAs’ everyday work (e.g., soothing patients, coping with mistakes, fostering team cohesion, or promoting apprentices’ education) as well as different conditions for a successful use of humor (e.g., positive social basis, current well-being, and social sensitivity). Further, the results suggest that the use of a certain humor style depends on the interlocutor as well as the intended goal of the humor. The results not only stress the multifaceted potential as well as the relevance of conditions of successful humor for MAs’ everyday work and education, but also provide valuable real-life insights into MAs’ everyday humor, thus offering several implications for practice to promote humor as a positive resource in MAs.

**Supplementary Information:**

The online version contains supplementary material available at 10.1007/s41042-022-00074-2.

## Introduction

There is robust evidence that humor has positive effects in various life contexts, such as improving private life (e.g., romantic relationships; Hall, 2017), working life (e.g., job satisfaction, motivation; Mesmer-Magnus et al., 2012), or education (e.g., student learning; Savage et al., 2017). Moreover, studies have shown that self-directed activities targeting humor (e.g., McGhee, 2010; Wellenzohn et al., 2016) increase happiness and resilience and, in positive psychology, humor is even considered as a morally positively valued trait (i.e., a character strength; Müller & Ruch, 2011; Peterson & Seligman, 2004). While humor research is an ever growing field that generates more and more interest, some areas are understudied. For example, there is only limited knowledge on the impact of humor in specific occupations. We aim at narrowing this gap in the literature by studying a particular group of healthcare professionals (HCPs): Medical Assistants (MAs) in Germany. Thus far, research suggested that HCPs may benefit from using humor by better coping with problematic situations and to foster positive social relationships (Dean & Major, 2008; McCreaddie & Wiggins, 2008; Wanzer et al., 2005). However, it has mainly focused on nurses and physicians, while paying little attention to other HCPs, such as physiotherapists (Thomson, 2010) or dentists (Nevo & Shapira, 1989). In this initial study we aim at learning more about how MAs use humor in their everyday work life and whether they see any benefits from using it.

In Germany, becoming a certified MA requires passing a three-year vocational education and training (VET); this means that MA apprentices learn through both, in–company training and a vocational school. Having passed VET successfully results in a recognized vocational qualification that qualifies for the work as an MA (Bundesinstitut für Berufsbildung, 2009). MAs work primarily in doctors’ practices or in hospitals, supporting physicians in their everyday work by performing clinical (e.g., drawing blood) and administrative tasks (e.g., appointment scheduling). MAs are the first contact person for patients in need of help; this makes them the “glue” for the healthcare system in several countries (Taché & Hill-Sakurai, 2010; Chapman et al., 2015; Erstling et al., 2019). In Germany, given that the COVID-19 pandemic strongly increased the number of worried patients turning to their primary care physician, MAs have become even more vital (Schnitzler et al., 2021). With MAs having such a core role in the healthcare system, the absence of research on the use of humor in their work is surprising. Using a qualitative approach, this study extends the literature on the usage and perceived functions (i.e., implicit beliefs about functions) of humor in HCPs by investigating its potential, the conditions for a successful use of humor as well as the role of humor styles.^1^

## State of Research on Humor in Healthcare and Purpose of the Current Study

### MAs’ Need for Resources and the Potential of Humor for HCPs

As previously discussed, MAs play a crucial role in the healthcare system in several countries (Chapman et al., 2015; Erstling et al., 2019; Schnitzler et al., 2021). Therefore, keeping MAs’ well-being and work performance high—to help them avoid mental illnesses or quitting (e.g., due to burn-out or poor work relationships)—is essential (Dreher et al., 2021; Vu-Eickmann et al., 2018).

The Job Demands-Resources (JDR) model posits that achieving this goal calls for job demands to be low and job resources to be high (Demerouti & Bakker, 2011). However, recent studies indicate that, for German MAs, job demands are very high; especially, the workload (e.g., time pressure, too many patients) and job control is typically low (e.g., due to interruptions and multitasking; Viehmann et al., 2017; Vu-Eickmann et al., 2018; Zaroti, 2015). Moreover, Dreher et al. (2021) surveyed more than 2,000 German MAs and show that COVID-19 led to the addition of newer demands (e.g., high risk of infection, uncertainty about the temporal scope of the crisis). Although Vu-Eickmann et al. (2018) found that MAs report having some resources (e.g., diversity of activities, patient interaction), they stress the importance of exploring and promoting more resources (Chapman et al., 2015; Dreher et al., 2021; Vu-Eickmann & Loerbroks, 2017). Furthermore, as the few studies on German MA apprentices indicate high job demands and high turnover for MA apprentices—who are in vocational education and training (i.e., VET) — exploring potential resources not only for experienced MAs but also for MA apprentices is critical (Schnitzler et al., 2021; Wiethölter, 2012).

One potential resource could be humor. Summarizing the literature on humor in healthcare there may be three ways humor can function as a resource in HCP work. First, humor serves as a tool for coping with stress, negative emotions and problems at work (e.g., death, illness, problematic patients), thereby also potentially improving the HCPs’ mental health (Linge-Dahl et al., 2018; McCreaddie & Wiggins, 2008; Wanzer et al., 2005). Second, humor is a tool for building and maintaining trustful and coequal relationships either with patients or within a working team, thereby increasing patient cooperation and positive teamwork (Dean & Major, 2008; Linge-Dahl et al., 2018; Proyer & Rodden, 2020). Third, humor serves as an educational tool improving, for example students’ attention, critical thinking, and emotional intelligence (Baid & Lambert, 2010; Bennett, 2003; Chabeli, 2008). However, as everyday working requirements of HCPs differ, the perceived functions of humor differ, too. Regarding interactions with patients for example, physicians use humor to reduce hierarchies and for counseling (Berger et al., 2004; Zayts & Schnurr, 2011) while nurses use humor to help patients cope (e.g., with illness and pain) or to comfort them (Dean & Gregory, 2005; Ji-Min & Hyunjoo, 2015). Moreover, most of the literature on humor functions in healthcare focuses on hospital and palliative care settings, lacking studies in primary care and practice settings (e.g., MAs’ context).

Thus, given that both MAs and MA apprentices have a high need for resources, and given that humor serves as a broad resource for other HCPs, researchers need to investigate functions of humor for MAs and their education. Research findings on humor as a resource for other HCPs likely serve as a starting point. But with humor being context specific and with working and educational contexts of HCPs differing, findings for nurses and physicians are not necessarily applicable to other HCPs in general and to MAs in particular (McCreaddie & Wiggins, 2008).

### Conditions of Successful Humor and the Role of Humor Styles in the HCP-Context

As humor might also have negative outcomes (e.g., feeling laughed at and therefore humiliated or excluded), it is crucial to investigate not only functions of humor but also conditions under which humor can be valuable for healthcare (Grainger, 2004; Platt et al., 2016; Schweikart, 2020). By summarizing the literature on *humor* in healthcare, we identify three main conditions that facilitate a successful use of humor (i.e., eliciting only positive reactions in everyone present as well as reaching the goal of the humor). First, there must be a positive social basis between the interlocutors (e.g., trustful relationship and a safe environment; Almeida & Nunes, 2020; Dean & Gregory, 2005; Siegel, 2005). Second, strong negative emotions must not be involved (e.g., highly anxious and stressed-out patients or recent loss; Bennett, 2003; Jones & Tanay, 2016; Pinna et al., 2018). Third, HCPs must possess social sensitivity to assess if humor is appropriate (e.g., empathy, identifying emotions; Dean & Major, 2008; Ji-Min & Hyunjoo, 2015; Olsson et al., 2001). However, most studies dealing with conditions under which humor can be valuable in healthcare either are non-empirical or mention but do not focus on these conditions. Moreover, these studies do not consider the role of different humor styles (e.g., benevolent humor vs. sarcasm/cynicism) that might require different conditions to be successful.

Yet, distinguishing between different humor styles — primarily between light (e.g., benevolent humor and fun) and dark styles (e.g., sarcasm and cynicism) is essential. Meta-analyses show that light styles correlate positively with mental health (e.g., optimism, well-being), whereas dark styles mostly correlate negatively (Jiang et al., 2020; Schneider et al., 2018). Also, they might contain exceptions to the rule: For example, some probably must not be based on a positive social interaction between the interlocutors (e.g., sarcasm). In healthcare, the appropriateness of HCPs’ use of dark humor, in particular, has long been an ambivalent topic as it helps HCPs to cope (e.g., with problematic patients) and foster team bonds and identification, but on the long term might dehumanize healthcare and demoralize HCPs (e.g., by creating prejudices and barriers; Aultman, 2009; Berk, 2009; Wear et al., 2009). Thus, some authors conclude that dark humor is “functional shorthand” (Piemonte, 2015, p. 384) but overall maladaptive.

Further, studies also show that the appropriateness of the usage of dark humor depends on how *dark humor* is defined. For example, whereas sarcasm and cynicism aim at humiliating people, gallows humor aims at humiliating tragic events and their symptoms (e.g., death, pain), making gallows humor an accepted coping resource (Aultman & Meyers, 2020; Watson, 2011). Further, a recent study with 104 nurses shows that aggressive humor (i.e., humor at others’ expense) correlates negatively to mental health, whereas self-deprecating humor (i.e., humor at one’s own expense) correlates positively (Navarro-Carrillo et al., 2020). Therefore, researchers who explore humor styles in healthcare need to define dark humor carefully and distinguish between specific dark styles. However, literature on humor styles in healthcare is still scarce, let alone studies that use an established humor style framework that distinguishes between different light and dark styles like the *Comic Style Markers* (CSM) by Ruch et al. (2018). The CSM comprise fun (good-natured jesting), wit (clever and spontaneous word plays), benevolent humor (tolerant, gentle and forgiving view on weaknesses and mistakes), nonsense (going beyond logical boundaries), irony (saying the opposite of what is meant that is only understood by insiders), satire (criticizing inadequacies with the aim to improve them), sarcasm (critical, biting remarks and *Schadenfreude*) and cynicism (comments that question morality and hypocrisy; CSM definitions by Hofmann et al., 2020).

### The Current Study

As the previous sections not only stressed the potential value of humor as a resource for MAs, but also highlighted the relevance of conditions for successful humor as well as the role of humor styles, this study explores MAs’ use of humor in everyday work and education with the following three main objectives. By applying a qualitative approach this study investigates (a) the perceived functions of MAs’ use of humor as a resource for MAs to master everyday work successfully; (b) the perceived conditions under which humor can be valuable for MAs; and (c) the role of different humor styles described in the CSM model (Ruch et al., 2018).

## Method

As to our knowledge, there is no study on the role of humor for MAs’ work and education to date, we applied a primarily explorative approach, gathering qualitative interview data close to MAs’ everyday use of humor (i.e., real-life examples of humorous situations) thus confirming ecological validity of the construct of *humor* in the MA-context.

### Recruitment and Sample

The study was conducted in Germany with German MAs. We included persons currently working as an MA – full time or at least 75% of a full time equivalent – having at least one year working experience. As investigating the functions of humor in MAs’ VET (i.e., Vocational Education and Training) was part of the research aim, we also sought for MA apprentices and favored MAs, who formerly or currently worked with MA apprentices. Recruitment ran from May until September 2020 via several channels (e.g., inquiries in doctors’ offices, personal contacts, social media).

We finally interviewed 14 German MAs aged 20–42 working in doctors’ single or group offices within teams of two to ten MAs, except for one MA being the only MA in a psychiatric practice and another MA working as head of 40 MAs in an ambulatory healthcare center. Interviewees were all female. However, as women are strongly over-represented in this occupation (Kathmann & Dingeldey, 2013), the findings of this study should be representative for this occupation regarding gender. MAs worked in different medical fields (e.g., general medicine, orthopedics and pediatrics; Online Resource 1). In order to – at least partly – control for possible COVID-19-induced changes of MA-working conditions that could have influenced humor instances we ensured that none of the MAs worked with severe COVID-19 patients. While twelve participants had at least three years MA-job-experience, two participants were still MA apprentices, both in their second year of VET. Further, nine of the experienced MAs currently or at least formerly worked with apprentices. At the beginning of the interview, we made sure that apprentices did the same daily work-related activities as the experienced MAs to ensure comparability across statements on humor.

### Data Collection

We conducted an individual interview with each of the recruited 14 MAs (July to October 2020). Due to pandemic-induced restrictions on gatherings, interviews were conducted via telephone. Further, as two MAs had highly restricted time capacity, they provided us their answers to interview questions in written form. All participants were provided with information on data privacy and gave written consent to data gathering and processing. Interviews were conducted in German (selected quotes are translated into English by the author) and took on average 90 min – including warm-up questions (regarding e.g., job-tasks and working climate).

All interviews were half-standardized and followed an interview guideline with the main topic *The Role of humor in everyday work and in-company training of MAs*. While some questions were standard in every interview (see below), we asked individual questions depending on the process and content of each interview (e.g., follow-up questions if the MA talked very emotionally about a specific humorous situation). Mandatory questions included (translated into English; for the full interview guide see Online Resource 2):

- Can you tell me about humorous situations in your everyday work? Give some examples!
- Imagine a new MA apprentice is beginning his/her VET in your practice tomorrow – What advice would you give her/him to take along regarding the use of humor?
- What role does humor play for your work as an MA?

Conducting the interviews in a half-standardized way allowed (a) comparability between the interviews and at the same time (b) individual requests that motivated MAs to share even more humorous situations. We audiotaped and transcribed the telephone interviews with the help of a research assistant who followed transcription rules (e.g., verbatim transcription and noting if the MA laughed or chuckled during the interview). Moreover, four of the interviewees additionally journaled situations in which humor occurred at work for two weeks. These journal entries served as additional data that we analyzed the same way as the interviews.

### Data Analysis

As intentions of the study were both, exploratory (i.e., exploring concrete functions and conditions) and theory-driven (i.e., applying the CSM by Ruch et al., 2018), we used qualitative content analysis with a combination of inductive (i.e., data-driven) and deductive (i.e., theory-driven) coding to analyze the data. In particular, we followed the variable-oriented approach by Schreier (2012) that enables analyses of frequencies and overlaps of codes allowing not only to gather (a) qualitative insights into functions and conditions of MAs’ use of humor, but also (b) evidence on their respective relevance as well as on the relation between functions of humor and humor styles. For the analysis, we used the software MAXQDA (Kuckartz & Rädiker, 2019).

The process of data analysis had six steps. Firstly, we read all interviews to get an overall impression on the role of humor in MAs’ everyday work. Secondly, we deductively created the two separate main codes *Functions of humor* and *Conditions of successful humor* and inductively created a subcode every time we found a new distinct humor function/condition in the data. The functions/conditions either were named by the MA (e.g., MA states “humor reduces anxiety”, MA#5) or emerged indirectly (e.g., MA talks about using humor in front of a patient whose anxiety thus decreased; or MA spontaneously uses humor in the interview to cope with aspects of her work). When suitable, we subsumed emerging functions/conditions under already existing codes to attain similar abstraction levels. Further, an *other/unclear* code was created to prevent forcing a coding decision in the following steps. Moreover, we analyzed both shared humor (i.e., humorous remarks in a conversation) as well as imagined humor (i.e., humor that only occurred in the MA’s head). The process of creating codes resulted in two coding systems (i.e., *Functions*/Table 1 and *Conditions*/Table 2). Thirdly, we conducted two coding trials with psychology students to validate the coding systems. Both systems – including a revision of *Functions* taking into account the disagreements of the first rating – yielded high inter-rater-reliabilities (IRR; *Functions*: Fleiss’ Kappa = 0.77, *z* = 35.57, *p* < 0.001; *Conditions*: Fleiss’ Kappa = 0.88, *z* = 18.13, *p* < 0.001; 4 raters; Landis & Koch, 1977; Sim & Wright, 2005). Fourthly, we coded the data using the two final coding systems. Every segment containing a humor function/condition was dedicated to only one subcode of the respective system.

**Table 1:** Perceived functions of MAs’ use of humor as well as occurrence of humor styles

Perceived functions of MAs’ use of humor in situations …	Coding frequency^a^	Humor style frequency^b^ (excluding unclear-code)		
		**Light**^**c**^	**Dark**^**d**^	**Ambivalent**^**e**^
**… within the team (or when MA is alone)**				
Cope with demands or shortcomings of patients	61	14	36	11
Foster team cohesion	45	12	8	13
Cope with own or colleague’s shortcomings	40	16	16	5
Cope with everyday work in general	30	4	3	5
Cope with team conflicts or disagreements	29	3	18	4
Cope with high workload	16	2	3	5
Reduce hierarchies	15	5	2	2
**Total**	**236**	**56**	**86**	**45**
**… with patients**				
Help Patients cope with illness or medical procedures	40	14	0	19
Build a connection to patient	35	14	4	10
Keep face in terms of own or work-related shortcomings	26	20	2	4
Educate and convince patients	22	5	4	13
**Total**	**123**	**53**	**10**	**46**
**… with apprentices**				
Take away apprentices’ fears and encourage them	19	9	2	2
Integrate apprentices into the practice team	19	10	2	2
Educate and convince apprentices	17	3	0	13
Set apprentices an example of using humor	13	2	3	1
**Total**	**68**	**24**	**7**	**18**
**Total Overall**	**427**	**133**	**103**	**109**

Notes: ^*a*^frequency of segments in which the respective function of MAs’ use of humor emerged; ^*b*^frequency of overlaps of respective *function*- and *style*-segments; ^c^ benevolent humor & fun; ^d^ sarcasm & cynicism; ^*e*^wit, nonsense, satire, irony, & gallows humor

**Table 2:** Perceived conditions of MAs’ successful use of humor

Condition	Coding frequency^a^	Example
A (positive) social basis	62	*“You have to know your patients; you know them at some point. You know, who likes what kind of humor and who does not. And this can help you (when using humor)” (MA#11)*
Humorous mood	48	*“When they (children) are so terrified that they won’t react to any joke, then you can tell jokes as long as you want but they are still terrified” (MA#14)*
Social sensitivity	35	*“You can tell what is appropriate in a situation (…) I have a gut feeling for this, like “Ok, everything is fine” or you see the sweat on the forehead, then you know “Ok, it’s not that relaxed, right now” (MA#7)*
Enough time and interactions	25	*“You were HUSTLING all day long and there was no time for anything funny (…) whatever kind of humor, there simply was no time” (MA#10)*
Going step by step	19	***“****I would recommend (…) to first watch the people and wait. When you talk to a patient and suddenly (…) HE says something funny himself, then you start to understand the patient better (…) so I go step by step” (MA#5)*
Matching nonverbal communication	18	*"(Sarcasm) can have a serious message, but with the pitch of your voice, the phrasing and with a wink of your eyes you can convey that you don’t want to shame someone, but to make aware of a mistake*” (MA#2)

Notes: ^*a*^Frequency of text segments in which a condition for MAs’ successful use of humor emerged

Fifthly, we deductively created a coding system for humor (comic) styles (i.e., *Styles*), using the descriptions of the CSM (Ruch et al., 2018) – which are on a relatively low abstraction level and close to everyday behavior – and further analyzed and coded each *Function*-segment regarding its prevailing comic style. When style was unclear, we coded *Unclear*. Applying such a validated framework that also contains a quantitative assessment instrument facilitates potential future research aiming at validating qualitative findings on the use of comic styles and their relation to functions of humor in the MA-context, or healthcare in general. In the result tables we will summarize the styles into *light* (i.e., benevolent humor, fun); *dark* (i.e., sarcasm, cynicism); and *ambivalent* MA-specific humor styles (i.e., nonsense, wit, irony, satire); this is based on the factorial structure of the CSM as well as suggestions on HCP-specific applications of the CSM (Hofmann et al., 2020; Proyer & Rodden, 2020; Ruch et al., 2018). Having an ambivalent category in this particular context reflects that these styles can have positive and negative effects and can be used for positive (e.g., coping, strengthening in-group cohesion) and negative (e.g., belittling) purposes in the daily work of MAs. Sixthly, using the Code-Relations-Browser of MAXQDA, we analyzed the frequencies of code overlaps between *Functions* and *Styles* to investigate relationships and patterns.

## Results

The main coding phase of all 14 interviews resulted in 674 coded segments (427 = *Functions* 207 = *Conditions;* 40 = *other/unclear*). Overall, every interviewee talked about humor as a positive and essential aspect of everyday work (e.g., “Without humor, you couldn’t do this job for long”, MA#11; “Humor is essential for survival”, MA#3) that might increase well-being of patients as well as MAs’ work satisfaction, motivation, and performance. Two MAs who stated that humor in their team was rather scarce – and who provided significant less humor examples, respectively – also stated that working climate in their practice is bad. However, depending on the situation and the interlocutor, humor might also have negative consequences and thus requires certain conditions to be successful.

### Perceived Functions of MAs’ Use of Humor in their Everyday Work and Education

In sum, 15 perceived functions of MAs’ use of humor emerged (Table 1). MAs spontaneously differentiated between humor (a) within the MA team (incl. when MA is alone); (b) with patients; and (c) with apprentices (i.e., VET).

#### Humor in Situations within the Team

Regarding team-situations, seven distinct functions of MAs’ use of humor emerged (*N* = 236), from which five were coping functions and two were social functions. Regarding coping, MAs used humor to cope either with everyday work in general (*n* = 30; e.g., “With humor I can handle and frame this better” – MA#2) or with specific work strains. Mostly, MAs used humor to – without patients’ knowing – cope with demands or shortcomings of patients (*n* = 61; e.g., calling overly demanding patients “little bloodsuckers” – MA#4; or commenting on wrong assumptions about medical topics with “Lord, send brain from heaven” – MA#11). Further, MAs reported using humor to cope with own or colleagues’ shortcomings (*n* = 40; e.g., commenting on having forgotten something with “Well, apparently styling my hair was more important” – MA#2) and conflicts (*n* = 29; e.g., placing a dancing Santa-Clause figure on the counter to signal truce – MA#7). Finally, MAs used humor to cope with high workload (*n* = 16); for example, by comparing the practice to a “call-center “ (MA#12) or by doing fictional yoga poses to relax (MA#10).

Regarding social functions, MAs, on the one hand, used humor to foster team cohesion (*n* = 45). By using in-jokes or running jokes (e.g., talking in funny languages) or by pranking a colleague (e.g., hiding a stinky cheese under the counter) they created “a feeling of cohesion” and “mutual memories” (MA#5). On the other hand, MAs stated using humor to reduce hierarchies (*n* = 15) to their supervisors (e.g., physicians). Humor – provided that it is bilateral – brings MAs “on eye level” with their supervisors (MA#5). However, this function emerged relatively rarely in the MAs’ narratives, probably because MAs have a rather “professional” (MA#9) relationship with their supervisors.

#### Humor in Situations with Patients

In situations with patients (*N* = 123), MAs’ use of humor showed four different functions. First, MAs used humor to help patients cope with illness or medical procedures (*n* = 40). MAs commented, for example, on a broken leg (e.g., “Anyway, you still have another one, right?” – MA#14), or an unpleasant mammography screening (e.g., “Imagine that the apparatus (….) just wants to hug you very tight” – MA#9) to soothe patients and to reduce their physical tension, in turn facilitating working with the patient. Second, MAs reported using humor to build a connection to patients (*n* = 35). MAs, for example, commented on patients’ (a) characteristics (e.g., comparing a patient’s blood sample with the red color of his shirt – MA#2) or (b) mishaps (e.g., commenting a patient offering her credit card rather than her insurance card with “Well, for this one I need the PIN, too!” – MA#13) to approach patients individually, thus keeping humanity in a medical world and increasing patients’ compliance.

Third, MAs used humor to keep face in terms of own or work-related shortcomings (*n* = 26). MAs commented, for example, own mishaps (e.g., after dropping something “Thank God, it didn’t fall upwards – otherwise I would need a ladder” – MA#10) or malfunctioning devices (e.g., “The computer needs some cuddles” – MA#7) to keep face and thus calm the waves, preventing patients from getting disgruntled. Fourth, MAs used humor to educate and convince patients (e.g., starting a specific type of intervention recommended by a medical doctor; *n* = 22). MAs humorously clarified wrong assumptions about medical issues (e.g., convincing an old patient to go to the hospital, otherwise his “legs will abandon” him – MA#2) or pointing out patients’ misbehavior (e.g., making a male patient aware of standing too close to another male patient by saying “If this is not your husband, please keep some distance” – MA#8).

#### Humor in Situations with Apprentices (i.e., in VET)

VET-specific situations with apprentices emerged less often than patient- or team-situations (*N* = 68), probably because (a) five interviewees never worked with apprentices and (b) talking about humor in one’s own apprenticeship needs retrospection. Still, four functions of MAs’ use of humor emerged.

First, MAs used humor to take away apprentices’ fears and to encourage them (*n* = 19). In particular, MAs used humor to reduce apprentices’ fears of making mistakes (e.g., commenting on a too firm bandage with “At least, the bandage would have lasted the whole day” – MA#13) to reduce apprentices’ insecurity and to encourage them to keep learning. Still, half of the interviewees stated that during their own apprenticeship the experienced MAs “made fun of the apprentice’s mistakes” (MA#3) instead of helping the apprentice to cope. Second, MAs stated using humor to integrate apprentices into the practice team (*n* = 19). MAs for example used little pranks or teasing (e.g., teasing an apprentice for losing his drivers’ license – MA#7) or integrate apprentices into team-insiders (e.g., insider of ironically commenting on one’s own mistakes – MA#2), to give them a feeling of being an appreciated team member and to establish a trustful relationship to the apprentice.

Third, MAs used humor to educate and convince apprentices (*n* = 17). MAs for example used creative humorous terms to help apprentices to memorize medical facts or taboos (e.g., “Don’t be a registration shrew” – MA#4) or they humorously comment on apprentices’ shortcomings to prevent (further) mistakes (e.g., “If you want to KILL the patient, you may try drawing blood from his wrist” – MA#11). The fourth function *Set apprentices an example of using humor* (*n* = 13) does not describe humor as a method facilitating VET – as the other three functions – but as training content (e.g., teaching how to use humor to reduce patients’ fears or how to tell if humor is appropriate). However, MAs who talked about humor as VET-content emphasized that simply telling the apprentices is not enough and you should rather show them in terms of a role model.

### Conditions of MAs’ successful Use of Humor

Every interviewee emphasized that a successful use of humor requires specific conditions to prevent negative reactions of interlocutors and to increase the probability of the intended outcome of the humor (e.g., reactions like laughing, smirking, disclosing personal information instead of making a long face and ignoring or criticizing the MA’s humor). In sum, six conditions for a successful use of humor emerged (Table 2). First, the MA and her interlocutor (e.g., patient, colleague) should have a (positive) social basis (*n* = 62). Knowing one’s counterpart’s “experiences and mentality” (MA#11) and especially her/his “type of humor” (MA#4) increases the probability of humor success. Moreover, having “the right chemistry” (MA#8) is even better than just knowing one’s counterpart. Second, the interviewees emphasized that everyone involved in the humorous situation should be in a humorous mood (i.e., mood to interact humorously; *n* = 48). Regarding situations with patients, MAs emphasized not to use humor in high emotional situations (e.g., fear of losing a child due to a febrile seizure – MA#14; or anger as one is not getting ones preferred medicine – MA#4). Regarding team-humor, on the other hand, “to spread humor in (a) practice the employees must be happy” (MA#12); for example, through the fulfilment of basic needs like appreciation and autonomy (MA#2).

Third, MAs highlighted the importance of social sensitivity (*n* = 35; i.e., “gut feeling” – named by four MAs; or “tact” – MA#9) to assess if humor is appropriate. This includes detecting verbal and nonverbal “signals” (MA#9) like facial expressions (e.g., smiling and eye-contact) and posture (e.g., open and approachable) as well as empathizing with interlocutors (e.g., their needs and emotional cognition) to assess if someone is in a humorous mood. Fourth, MAs need enough time and interactions (*n* = 25). As in “acute and stressful situations” (MA#14) humor is scarce, MAs need breaks like “five minutes at the end of a team meeting” (MA#5) or “breathers” to “keep (their) humor” (MA#4). Moreover, phases when MAs work alone (e.g., sort patient files) or talk to patients on the phone instead of face-to-face, do not provide enough possibilities to use humor; at least those types that require social interactions, while humorous ideas or thoughts would probably be observable.

Fifth, MAs should go step by step when applying humor (*n* = 19). MAs advised to first “check the situation, before getting out (the) joke collection” (MA#13) by “waiting and watching” how the other one acts (MA#5). MAs often started with a small talk (e.g., “Did you had a good way here?” – MA#5) and if the counterpart is talkative they “take it up” (MA#9) and “slide in with a funny commentary” (MA#10). Sixth, MAs should use matching nonverbal communication when using humor (*n* = 18), like facial expressions (e.g., smiling, winking or wide opened eyes) and pitch of the voice (e.g., sounding “extra naïve” or exaggeratory – MA#2). MA#13, for example, named two situations in which she did not get the irony, as her counterparts conveyed the humor “in such a serious way (…) being totally straight-faced”.

### The Occurrence of Humor Styles

Overall, MAs used all eight CSM; we decided to additionally code for *gallows humor* (e.g., Watson, 2011; Table 1 & Online Resource 3) as it seemed rather typical for MAs in the present study. Light (i.e., benevolent humor, fun) and dark (i.e., sarcasm, cynicism) humor – both containing two CSM – emerged relatively balanced (*n light* = 133 vs. *n dark* = 103), while ambivalent styles emerged relatively scarcely (*n* = 109), considering that this category contains five specific styles (i.e., wit, nonsense, irony, satire, and gallows). However, the frequency of humor styles differed regarding the type of interlocutor. For example, while MAs used dark humor in 46% of situations within the team, with patients and apprentices, dark humor was scarce (9%/14%).

Further, the number of examples given for each style differed between humor functions. In team situations, for example, when coping with patients’ demands and shortcomings and with team conflicts or disagreements MAs used dark humor in more than 50% of the segments, while when coping with shortcomings, light and dark humor were used equally. Two MAs described the dark humor in team-situations – mostly sarcasm – as “positive” (MA#10) or “benign” (MA#4) sarcasm, as – at least from their perspective – the interlocutor “understands it” and knows that “it is not meant like that” (MA#4). Regarding situations with patients, when helping patients cope and when educating or convincing patients, MAs mostly used ambivalent styles (coping = 58%, especially wit and gallows humor; educating = 59%, especially satire). When keeping face in terms of own or work-related shortcomings, on the other hand, light humor prevailed (77%, especially benevolent humor). Further, when taking away apprentices’ fears and when integrating apprentices into the practice team, light humor was dominant (taking away fears = 69%, especially benevolent humor; integrating = 71%, especially fun), while when educating and convincing apprentices, ambivalent styles prevailed (81%; especially wit and satire).

However, although in most perceived functions certain humor styles prevailed, other styles also occurred to relatively high amounts (e.g., when helping patients cope, ambivalent styles prevail (58%), but closely followed by light humor; 42%). Moreover, as absolute coding frequencies (a) differed between functions and (b) were relatively low for some functions (especially, in situations with apprentices), the findings should not be over-interpreted.

## Discussion

The interviews offered valuable initial insights into MAs’ use of humor in work and education, specifically providing insights into (a) distinct perceived functions of MAs’ use of humor in different situations (i.e., within team, with patients, with apprentices), (b) conditions for humor to be successful and (c) the occurrence of different humor styles and their relation to humor functions. The key findings are that MAs actually perceive a broad range of functions of humor in their working life and that they employ a variety of humor styles, with those being more positive and encouraging more frequently mentioned. However, interactions concerning the situation were noticeable; namely, that with patients and apprentices MAs state to use mostly light (especially benevolent humor) and ambivalent (especially wit) styles, whereas within the team they use mostly dark humor (especially sarcasm). Further, MAs perceive several essential conditions for humor to be successful and to reach its aim in the interaction with patients and colleagues.

### Coping—Social—Educational: Functions of MAs’ Use of Humor are multifaceted

Data analysis revealed various perceived functions of MAs’ use of humor. For example, MAs used humor to (a) cope with different work strains (e.g., shortcomings of patients, conflicts); (b) foster social cohesion and relationships; and (c) educate and convince others. This relates well to the three main functions of humor in healthcare, which we identified during literature analysis (i.e., coping – McCreaddie & Wiggins, 2008; social – Dean & Major, 2008; educational – Chabeli, 2008). However, these three main functions have different aims and outcomes depending on the type of interlocutor. For example, while in team-situations, MAs reported using humor rather as a valve for their own negative emotions (e.g., letting out anger due to problematic patients), in situations with patients, they used humor to help patients relax (e.g., due to painful treatment). Further – regarding the social function – in team-situations, MAs suggested that humor strengthens cohesion, while with patients they argued that humor has the potential to improve patients’ compliance and helps to keep humanity. In situations with apprentices, on the other hand, they indicated that humor helps making apprentices feel an appreciated team member. When coding the examples provided, it was evident that some functions cannot be as easily assigned to one of the three main functions. For example, when keeping face in terms of own or work-related shortcomings, MAs use humor to mitigate the shortcoming (i.e., coping) and simultaneously keep a good impression (i.e., social). A study of humor in palliative care identified a similar function called *express dignity* (Dean & Gregory, 2005).

Further, the topic *coping with peoples’ shortcomings* (e.g., mistakes and misfortunes) stands out in particular. MAs reported using humor to cope with own, patients’ and colleagues’ shortcomings and they help apprentices to cope with their shortcomings. However, half of the interviewed MAs reported negative experiences regarding dealing with mistakes during their own apprenticeship (i.e., experienced MAs made fun of them). Some of them even wish that their current team would cope more humorously with colleagues’ mistakes. In the literature on HCPs’ use of humor, coping with peoples’ mistakes and misfortunes rarely is a distinct function (e.g., the function *mitigating own errors* by Archer et al., 2019). Therefore, MAs should try to increase their use of benevolent humor when coping with colleagues’ and, especially, with apprentices’ mistakes to create a positive error culture in the long-term.

Moreover, despite potential recall biases (e.g., memorizing and reporting specific situations), coding frequencies provide some suggestions on the relevance of the perceived functions of MAs’ use of humor. For example, humor was mostly used for coping functions (*N* = 235), second most used for social functions (*N* = 114) and least used for educational functions (*N* = 39). Even in VET-situations only 25% of segments were dedicated to the function *educate and convince apprentices*, while in 75% of segments humor served other functions (e.g., encouraging or socializing). These findings support former studies on humor in medical education, which found similar functions and relevance distributions of functions (e.g., humor fosters learning but also, and more often, relaxes students; Chabeli, 2008; Liu et al., 2017), but on the other hand, also add insights into the relevance of certain educational functions. Overall, the identified functions of MAs‘ use of humor support the literature on functions of HCPs’ use of humor (i.e., coping, social and educational), but also provide new insights regarding further functions (e.g., coping with shortcomings) as well as concerning the potential relevance of functions (i.e., coding frequencies). For practice, in particular, the findings (a) suggest for which working requirements humor might be a valuable resource (e.g., adding a hint of humor when soothing patients, or when teaching apprentices) and (b) provide concrete examples for MAs’ use of humor (e.g., humorous phrases, words and behavior) for different occasions (e.g., within team, with patients).

### Conditions of MAs’ successful Use of Humor – Social Competencies as Key?

Data analysis revealed six distinct conditions MAs highlighted for preventing negative outcomes of humor (e.g., humiliating) and increasing the success of humor (e.g., relieving). Taking a look at the literature on conditions of successful humor in healthcare, it appears that the three most frequently coded conditions support the three main conditions that we found during literature analysis; namely, positive social basis (e.g., Dean & Gregory, 2005), avoiding high emotional situations (e.g., Jones & Tanay, 2016), and Social Sensitivity of HCPs (e.g., Ji-Min & Hyunjoo, 2015). However, regarding the second condition, MAs stated not only to avoid humor in highly emotional situations with patients, but to – in situations within the team – use humor only with colleagues who have a certain job-satisfaction and are thus in the mood to act humorously. As the remaining three conditions, emerge only scarcely in the – mostly non-empirical – studies on HCPs’ use of humor so far (e.g., Osincup, 2020; Siegel, 2005), they give additional insights into conditions of successful humor in healthcare. While *Enough time and interactions* suggests, that the occurrence of humor depends on working conditions (e.g., low workload and frequent face-to-face interactions), *Going step by step*, and *Matching nonverbal communication* give concrete behavioral advices for the application of humor (e.g., small talk before using humor and adapting pitch of the voice).

Further, comparing the six conditions, it appears that four of them might interrelate. Having a high social sensitivity and knowing how to communicate adequately might facilitate a careful step by step approach as well as building up a positive social basis to people. This suggests a common meta-condition like *social competencies* (Dietzen & Tschöpe, 2019). Indeed, several psychological studies find robust correlations between social competencies and humor styles (e.g., Amjad & Dasti, 2020; Yip & Martin, 2006). However, study designs rarely allow causal inference and humor is never operationalized as humor success (i.e., humor that leads to positive reactions and outcomes independent of humor style). Therefore, further studies need to investigate the causal relationship between social competencies and humor success, especially in HCPs.

Lastly, as literature on humor styles in healthcare shows that dark humor can humiliate and discourage people, one could expect light humor to be a condition for successful humor. However, in the data, conditions emerged during situations with light as well as dark humor. This suggests that both – light and dark humor – could be unsuccessful and that MAs face the difficulty to ensure specific conditions in both cases. Hence, person × situation-factors need to be considered. Taking into account that (a) humor has high potential for MAs and (b) social competencies might play a key role for successful humor, a combination of humor- as well as social competencies-trainings might be useful to help MAs to confidently use humor in an appropriate and helpful way. Findings provide suggestions for learning contents (e.g., application possibilities of humor like soothing patients; essential social competencies like emotion detection) as well as for successful transfer into everyday work (e.g., work-related humor examples; recommendations for supervisors how to enable the occurrence of humor at work). As MAs suggested that a certain level of MA-happiness is essential to engage with humor, especially McGhee’s (2010) training might be a suitable approach, as it aims at fostering a positive sense of humor gradually (i.e., preventing potential reactance) with the final step “finding humor in the midst of stress” (i.e., enabling MAs to find humor even if their level of happiness is rather low). For example, Linge-Dahl et al. (2022) recently ameliorated nurses’ self-reported distress levels and elevated mood by means of humor workshops based on McGhee’s approach. However, as there might always be gelotophobes (= people who fear being laughed at) among the participants, implementing such programs in practice must be done carefully (cf. Platt et al., 2016).

### MAs’ Use of Humor Styles is situation-specific: Is Dark Humor justified?

Frequency and overlap analyses show that the number of reported humor styles differed regarding type of interlocutor (e.g., more dark styles in the team than with patients or apprentices) and function (e.g., with patients: mostly light styles to keep face while mostly ambivalent styles to educate/convince patients). This suggests that MAs – maybe purposefully – choose using a certain humor style dependent on its suitableness and potential success in a situation (e.g., mocking styles might be riskier with (new) patients; benign humor might be most successful to reduce fears of insecure apprentices). It will be interesting to test in the future what implicit theories and learning experiences from past behaviors MAs use for selecting the styles and what cognitive processes inform such decisions—or whether other explanations (e.g., following one’s own preferred humor style) will explain usage of a specific humor style best.

Further, the relatively high amount of dark humor in situations within the team stood out – especially, the high frequency of reported sarcasm to cope with different strains (Table 1) needs to be highlighted. As sarcasm can have negative outcomes (e.g., humiliation, prejudices; see Aultman, 2009; Berk, 2009), one might recommend MAs to decrease their use of dark humor. For example, instead of mocking colleagues because of their shortcomings, MAs could apply more virtuous humor styles like satire to correct those shortcomings (Proyer & Rodden, 2020). However, interviewees emphasized positive attributes of sarcasm in everyday work (e.g., a valve to let off “steam”, part of the team culture), even naming it “positive/benign sarcasm” (MA#4 & MA#10). One explanation for these findings might be that interviewees are only aware of the short-term outcomes (e.g., relieving) of dark humor, and not its negative long-term outcomes (e.g., dehumanization and demoralization; see e.g., Piemonte, 2015).

However, as empirical studies on humor styles in healthcare are still scarce and as the findings of this study suggest that MAs see potential benefits of the usage of dark humor in the team-context, further studies investigating the benefits of dark humor for the team-context seem warranted. Moreover, this, as well as the finding that the occurrence of humor styles varies with functions, suggest the existence of certain humor style-combinations that might be optimal for the MA-context (e.g., preferring light humor, but using also dark humor now and then). The few existing empirical studies on the combination of humor styles suggest, for example, that dark humor styles are not – or at least less – damaging when used alongside positive humor styles (Evans & Steptoe-Warren, 2018; Leist & Müller, 2013).

### Limitations and Future Directions

While this study provides new insights into the potential of MAs’ use of humor, it also has some methodological limitations. First, as the sample consists of only (a) 14 and (b) German MAs, validity and generalizability of findings is restricted – especially for the humor functions in VET-situations, as only two apprentices participated and only few VET-functions could be coded. Having the COVID-19 pandemic challenging the German healthcare system heavily since March 2020 (i.e., increased workload of MAs), recruitment of MAs was difficult and was a hindrance for collecting a larger sample. Therefore, future studies should test the newly derived hypotheses (e.g., social competencies as crucial factor for humor success, the existence and benefits of different humor style-combinations) with larger and more diverse samples. Further, the a) uneven shares of experienced MAs versus apprentices and b) different ways of data collection (i.e., only written vs. only oral vs. oral and journal) might also have influenced the results (e.g., keeping a journal might have induced positive mood and thus awareness for especially positive humor instances; Wellenzohn et al., 2016).

Moreover, as the COVID-19-pandemic had a severe influence on HCPs’ well-being (Dreher et al., 2021) and might have influenced the occurrence of humor at the workplace (e.g., increased dark humor to cope with the uncontrollability), findings might be biased. Although, we tried to control this bias by excluding COVID-19-specific humor-statements and -examples (e.g., “One cough is not enough for quarantine” – MA#4), findings are probably still influenced (e.g., more dark humor like sarcasm). Further, all interviewees primarily talked about positive – and not negative – functions of humor. This may have different reasons like sampling bias (i.e., only MAs who have a positive view on humor), social desirability (i.e., not admitting using humor for antisocial purposes) or perception bias of the MA (e.g., patient perceives an illness and thus a joke on that illness as more severe than the MA does but – being dependent on the MA – laughs anyway). Therefore, future studies should explore potential negative functions of MAs’ use of humor (e.g., increasing one’s own status by belittling someone else) for example by interviewing patients and/or the MA’s colleagues on how they perceive the MA’s humor. Related to that, of course, there are other ways of operationalizing *successful humor* than those used in the present study. Therefore, follow-up studies could take additional variables into account (e.g., an analysis of language use or direct observations of facial expressions either when recounting humorous incidents or when being in a humorous interaction with a patient).

Further, despite the high IRR of the two coding systems, functions and conditions of humor might interrelate, respectively. For example, a humor-induced team cohesion might in turn increase the mutual – even humorous – coping with work strains (Smith et al., 2013). Still, these causal interrelations are probably not absolute and differentiating between the single functions allows the depiction of the manifold application possibilities of humor.

## Conclusion

This qualitative study with MAs from different medical fields gives crucial insights into the role of humor in MAs’ everyday work and education. MAs use humor with different interlocutors (e.g., colleagues, patients, or apprentices) for various coping-, social and educational purposes (e.g., coping with strain, connect with patient, and educate apprentices). This suggests that humor is a multifaceted resource for MAs. Further, findings emphasize the relevance of specific conditions for a successful use of humor (e.g., social sensitivity of MA, humorous mood of interlocutor) and indicate a situation-specific usefulness of different humor styles (e.g., using light humor to help patients cope but dark humor to cope with own strains). Although further studies need to examine the generalizability of these findings, this study provides a first framework of a positive and successful use of humor for MAs as well as various approaches for further research on humor in healthcare and MAs in particular.

## Supplementary Information

Below is the link to the electronic supplementary material.

Supplementary file1 (DOCX 19.1 KB)

Supplementary file2 (DOCX 25 KB)

Supplementary file3 (DOCX 18 KB)

## Data Availability

As the authors confirmed to the participants that no data will be shared with third parties, raw data is not available.

## Footnotes

1 Being aware of the differing labeling of different kinds of humor as well as the varying definitions of the term *Humor* (Ruch, 2008; Ruch et al., 2018), the authors want to clarify the use of the terms *Humor Styles* and *Comic Styles* in this study. The term *Humor Styles* means the general concept that humor (i.e., the comic/the funny) can have different qualities and can show in different forms. Thus, *Humor Styles* include frameworks like the here used *Comic Styles* by Ruch et al. (2018) but also other theoretical frameworks of styles or styles that occurred in explorative studies. Therefore, the *Comic Styles*, in this study, are the operationalization of *Humor Styles*.

## Declarations

The manuscript is not submitted for publication or considered for publication elsewhere.
