# Supplementary material for: Humor as a Multifaceted Resource in Healthcare: An Initial Qualitative Analysis of Perceived Functions and Conditions of Medical Assistants’ Use of Humor in their Everyday Work and Education
Source: Int J Appl Posit Psychol. 2022 Oct 13;7(3):397–418. doi: 10.1007/s41042-022-00074-2 (PMC9559119; doi:10.1007/s41042-022-00074-2)
Supplement: Supplementary file 1 — Supplementary file1 (DOCX 19.1 KB) [file 41042_2022_74_MOESM1_ESM.docx]

**Online Resource 1**

**Additional information on the interviewed female medical assistants**

| **Abbreviation for interviewed MA** | **Age** | **Medical Field** |
| --- | --- | --- |
| MA#1 | 27 | General Medicine |
| MA#2 | 40 | General Medicine |
| MA#3** | 29 | General Medicine |
| MA#4* | 32 | General Medicine & Infectiology |
| MA#5 | 42 | Orthopedics |
| MA#6** | 30 | Orthopedics & Trauma Surgery |
| MA#7 | 35 | Trauma-/emergency Surgery |
| MA#8 | 36 | Oncology |
| MA#9 | 26 | Radiology & Mammography |
| MA#10 | 33 | Urology & Urooncology |
| MA#11 | 25 | Gynecology |
| MA#12* | 20 | Pneumology & Allergology |
| MA#13 | 23 | Psychiatry & Psychotherapy |
| MA#14 | 35 | Pediatrics |

Notes: *MA apprentice; ** interviews in written form
