# Supplementary material for: Humor as a Multifaceted Resource in Healthcare: An Initial Qualitative Analysis of Perceived Functions and Conditions of Medical Assistants’ Use of Humor in their Everyday Work and Education
Source: Int J Appl Posit Psychol. 2022 Oct 13;7(3):397–418. doi: 10.1007/s41042-022-00074-2 (PMC9559119; doi:10.1007/s41042-022-00074-2)
Supplement: Supplementary file 2 — Supplementary file2 (DOCX 25 KB) [file 41042_2022_74_MOESM2_ESM.docx]

**Online Resource 2**

**Interview guideline regarding the role of humor in everyday work and in-company training of medical assistants (MAs)**

(Mandatory Questions = **bold**; Intro/Outro/bridge passages = *italic*)

*Thank you for conducting this interview with me! The topic of the interview is „The Role of humor in everyday work and in-company training of MAs”. In the course of this interview I will ask you several questions concerning this topic – please tell me everything that is relevant and important for you in this matter****!*** *Please feel free during talking – I will not judge anything you tell me. I am interested in YOUR experiences, thoughts and feelings. Do you have some questions beforehand?*

**First, can you please tell me something about your everyday work (and VET) in general?**

- What does a typical day look like in your everyday work?
- What is especially challenging/ positive in your everyday work?
- (For apprentices) What do you think about your in-company training/ your trainers?

**What is *humor* in your opinion? How would you define humor?**

- Why do you think so?
- What is the adding value compared to mere optimism/positivity?

**Can you tell me about humorous situations in your everyday work? Give some examples! (You can also relate to your humor journal)**

- What was the goal of the humor in this situation?
- What kind of effects/consequences did humor have in this situation?
- Can you remember a situation, where humor was inappropriate?
- How do you know, if using humor is appropriate/helpful in a situation?
- Do you wish for more humor in your practice?
- **Can you tell me about humorous situations especially with apprentices? Give some examples!**
  - **AND/OR (if never worked with apprentices OR interviewee is apprentice herself): Please recall your own apprenticeship: Can you tell me about situations when trainers used humor in your presence, addressing you in your role as the trainee?**

**Imagine a new MA apprentice is beginning his/her VET in your practice tomorrow – What advice would you give her/him to take along regarding the use of humor?**

**Please compare the time BEFORE and AFTER the beginning of Covid-19 – Are there differences regarding the use of humor in your everyday work?**

**Please reflect everything we talked about so far: What role does humor play for your work as an MA?**

- What function(s)/outcomes does humor have in your everyday work?
- What if there was no humor at all at your work?

*Now, I would like to ask you about specific humor styles. I will name eight humor styles and explain them in one sentence according to the literature.*

**Please rate each style regarding its occurrence as well as its appropriateness for your everyday work. Use a scale^[[1]](#footnote-1)^ from 1 (= never/not appropriate at all) to 10 (= very often/ totally appropriate), respectively.**

- **Fun:** jesting and/or playing tricks on somebody to spread good mood.
- **Irony**: saying the opposite of what is meant that is only understood by insiders and confuses outsiders
- **Wit**: Putting everyday incidences in a nutshell in a witty way. Others find it surprising and exhilarating; creative and witty comments/descriptions.
- **Sarcasm**: criticizing and mocking others, (e.g., through Schadenfreude) to show somebody up.
- **Benevolent Humor**: Commenting shortcomings of life in a humorous and benign way, therey including oneself; humorously and positively reframing negative things (e.g., the world is not perfect).
- **Satire**: criticizing inadequacies in a funny way with the aim to improve them (e.g. imitating someone’s behavior in a funny way to make clear to him/her that his/her behavior is wrong).
- **Nonsense**: playful, illogical humor that can also be spirited but has no goal/intention (e.g., ridiculous, silly, dancing/singing, talking in nonsense languages/dialects)
- **Cynicism**: mocking comments that question/criticize morality and virtues of others.

**Is there something else you want to tell me regarding humor in your everyday work that has not been mentioned yet?**

*Thank you very much for your time and for sharing all these experiences with me! If you are interested, I can send you the record and/or the transcript of the interview and/or later the results of the study.*

1. Due to restricted validity of the shortened descriptions of the humor styles (i.e., humor styles in the example-situations that MAs told to explain their ratings did not match the humor style that was to be rated) subjective, quantitative ratings were not analyzed. However, we analyzed the example-situations that MAs added during rating regarding the three research focuses (i.e., functions, conditions, humor styles). [↑](#footnote-ref-1)
