# Supplementary material for: Humor as a Multifaceted Resource in Healthcare: An Initial Qualitative Analysis of Perceived Functions and Conditions of Medical Assistants’ Use of Humor in their Everyday Work and Education
Source: Int J Appl Posit Psychol. 2022 Oct 13;7(3):397–418. doi: 10.1007/s41042-022-00074-2 (PMC9559119; doi:10.1007/s41042-022-00074-2)
Supplement: Supplementary file 3 — Supplementary file3 (DOCX 18 KB) [file 41042_2022_74_MOESM3_ESM.docx]

**Online Resource 3**

**Occurrence (i.e., frequency) of the eight comic style markers in situations within the team/when alone, with patients and with apprentices**

|  | **Bene-volent** | **Fun** | **Sar-casm** | **Cyni-cism** | **Wit** | **Non-sense** | **Irony** | **Satire** | **Gal-lows** |
| --- | --- | --- | --- | --- | --- | --- | --- | --- | --- |
| **Team/Alone** | 28 | 28 | 61 | 25 | 12 | 18 | 6 | 3 | 6 |
| **Patients** | 39 | 14 | 7 | 3 | 15 | 5 | 7 | 8 | 11 |
| **Apprentices** | 14 | 10 | 6 | 1 | 8 | 0 | 4 | 4 | 2 |
| **Total** | 81 | 52 | 74 | 29 | 35 | 23 | 17 | 15 | 19 |
